# Chickenpox Outbreaks in Three Refugee Camps on Mainland Greece, 2016-2017: A Retrospective Study

**DOI:** 10.1017/S1049023X23006702

**Published:** 2024-02

**Authors:** Sarah Elizabeth Scales, Jee Won Park, Rebecca Nixon, Debarati Guha-Sapir, Jennifer A. Horney

**Affiliations:** 1. Epidemiology Program, University of Delaware, Newark, Delaware, USA; 2. Department of Geography and Spatial Sciences, University of Delaware, Newark, Delaware, USA; 3. Division of International Health, Johns Hopkins University Bloomberg School of Public Health, Baltimore, Maryland, USA

**Keywords:** complex emergency, disaster epidemiology, displacement, humanitarian health, varicella

## Abstract

**Introduction:**

Displaced populations face disproportionately high risk of communicable disease outbreaks given the strains of travel, health care circumstances in their country of origin, and limited access to health care in receiving countries.

**Study Objective:**

Understanding the role of demographic characteristics in outbreaks is important for timely and efficient control measures. Accordingly, this study assesses chickenpox outbreaks in three large refugee camps on mainland Greece from 2016 – 2017, using clinical line-list data from Médecins du Monde (MdM) clinics.

**Methods:**

Clinical line-list data from MdM clinics operating in Elliniko, Malakasa, and Raidestos camps in mainland Greece were used to characterize chickenpox outbreaks in these camps. Logistic regression was used to compare the odds of chickenpox by sex, camp, and yearly increase in age. Incidences were calculated for age categories and for sex for each camp outbreak.

**Results:**

Across camps, the median age was 19 years (IQR: 7.00 - 30.00 years) for all individuals and five years (IQR: 2.00 - 8.00 years) for cases. Males were 55.94% of the total population and 51.32% of all cases. There were four outbreaks of chickenpox across Elliniko (n = 1), Malakasa (n = 2), and Raidestos (n = 1) camps. The odds of chickenpox when controlling for age and sex was lower for Malakasa (OR = 0.46; 95% CI, 0.38 - 0.78) and Raidestos (OR = 0.36; 95% CI, 0.24 - 0.56) when compared Elliniko. Odds of chickenpox were comparable between Malakasa and Raidestos (OR = 1.49; 95% CI, 0.92 - 2.42). Across all camps, the highest incidence was among children zero-to-five years of age. The sex-specific incidence chickenpox was higher for males than females in Elliniko and Malakasa, while the incidence was higher among females in Raidestos.

**Conclusion:**

As expected, individuals five years of age and under made up the majority of chickenpox cases. However, 12% of cases were teenagers or older, highlighting the need to consider atypical age groups in vaccination strategies and control measures. To support both host and displaced populations, it is important to consider risk-reduction needs for both groups. Including host communities in vaccination campaigns and activities can help reduce the population burden of disease for both communities.

## Introduction

Refugee populations are primed for negative health outcomes due to the stressors associated with displacement, transit, limited access to health care, and crowded and unhygienic living conditions.^1^ Following the closure of the Balkan Route in 2016, refugees who crossed the Mediterranean via unauthorized sea routes were severely limited in their ability to move beyond mainland Greece.^2–4^ To address the health care needs of refugees and support Greek civil services, the European Union’s Civil Protection and Humanitarian Aid Operations (Brussels, Belgium) supported Médecins du Monde (MdM; Saint-Denis, France) operations in camps throughout mainland Greece.^5^ Recognizing the potential negative health outcomes related to vaccine-preventable diseases in refugee populations, this study characterizes varicella outbreaks among refugees in terms of age, sex, and camp using MdM clinical line-list data from Elliniko, Malakasa, and Raidestos refugee camps. Outbreak characteristics are relevant given the proportion of adolescent refugees making Mediterranean crossings.^6,7^

### Circumstances in Countries of Origin

Varicella, or chickenpox, is a communicable disease caused by the varicella-zoster virus. Descriptive characteristics and risk factors for infection are under-studied in the Eastern Mediterranean Region, particularly in Afghanistan and Syria.^8^ Varicella vaccination is not included in Syrian and Afghan routine immunization schedules, and surveillance systems are not robust.^9^ In Afghanistan, outbreaks among adults are not uncommon. In 2010, there was an outbreak among recruits aged 18 to 44 years old from the Afghan National Civil Order Police in Herat, Afghanistan.^10^ Prior to the start of the Syrian Civil War, 91% of female students at a pharmacy school in Syria had immunoglobulins against chickenpox, attributed to community-level virus circulation and varicella immunization programs.^8,11^ However, the Syrian Civil War has led to the degradation of health care infrastructure within the country and compromised progress made toward wide-spread immunization coverage for vaccine-preventable diseases.^12,13^

Until April 2017, immunization activities among refugees in Greece were coordinated by non-governmental organizations.^14^ After April 2017, immunization activities were formalized through the PHILOS program, which was spearheaded by the Greek Ministry of Health (Athens, Greece) and the Hellenic Centre for Disease Control and Prevention (Athens, Greece). However, varicella was not considered a priority vaccine, likely due to the high level of need to increase immunization coverage. Population-level immunity against chickenpox among refugees in camps across mainland Greece was largely unknown (eg, no administrative coverage estimates) and likely dependent on pre-existing, infection-derived immunity.

A 2009 study found that nearly 22% of adolescents in Greece were susceptible to chickenpox and concluded that Greek adolescents with no prior infection and without household exposures should be eligible for presumptive varicella vaccination.^15^ Eastern Europe has a high prevalence of chickenpox, creating an additional layer of risk both for refugees in the region and host communities and health systems.^16^ The Mediterranean and Balkan transit pathways place both refugees and host communities at high risk for chickenpox outbreaks. Therefore, while immunization against high morbidity and mortality infectious diseases takes priority in resource-limited settings, varicella vaccinations should be considered in routine immunization activities for refugees in the region.

### Natural History of Varicella

Chickenpox is a vaccine-preventable disease caused by the varicella zoster virus. Prodromal symptoms such as malaise, headache, and fever, before the development of a rash;^17^ prodromal symptoms are more common as patient age increases.^18^ The incubation period of chickenpox is 15 days on average, with a range of 10 to 21 days.^19^ Cases are most contagious one-to-two days prior to rash onset and continue to be infectious for five-to-seven days after appearance.^20^ The reproductive number for chickenpox is roughly ten, meaning that an individual chickenpox case will, on average, infect ten additional susceptible individuals.^21^

### Seasonality and Climatic Drivers of Chickenpox

Chickenpox is typically considered an early childhood disease in temperate countries, with the majority of the adult population having immunity from prior infection or vaccination.^22^ However, age-specific immunity patterns are not concrete, especially when considering large-scale population displacement, shifts in population demographics, and changes in climatic drivers of disease dynamics.^23–25^ While it is widely accepted that there are differences in the natural history of chickenpox between tropical and temperate zones, these differences are attributed to a range of factors.^26,27^ In tropical climates, chickenpox has a more distinct seasonal pattern - with cases occurring in spring and winter - compared to temperate climates where cases occur throughout the year with lowest case counts during summer months.^24,28–31^ Across all climatic zones, temperature and rainfall contribute to the seasonality of disease.^32,33^ A modelling study of chickenpox dynamics in Mallorca, a Mediterranean island, found that solar radiation and water vapor pressure were climatic drivers of chickenpox seasonality, with peaks in case numbers in June.^29^ In a retrospective study of pediatric and adolescent chickenpox cases over a 22-year period in Athens, Greece, case numbers were inversely related with air temperature, peaking in May, dropping, and rising again in late autumn.^28^ Academic calendars and other drivers of higher contact rates, such as weather patterns, population density, mobility, and crowding, also contribute to the seasonality, as well as deviations from seasonality of chickenpox infections.^34,35^

### Relevance of Age- and Sex-Disaggregated Analyses

Age- and sex-disaggregated data analyses of communicable disease outbreaks, particularly in displaced populations, are important for timely and appropriate outbreak control measures. A study characterizing measles outbreaks in Dollo-Ado and Dadaab refugee camps in Ethiopia and Kenya found that immunization activities in the early stages of outbreak response were not targeting the age groups that were driving the outbreak.^36^ Similar to measles, the clinical severity of disease increases as chickenpox cases become older.^23^ A study in the United Arab Emirates found that adults with chickenpox had increased risk of severe clinical symptoms and complications such as pneumonia.^37^ In European Medicines Agency member countries, adults experienced higher morbidity and mortality due to chickenpox.^38^

The objective of this study is to not only present the scale of chickenpox outbreaks in refugee camps on mainland Greece, but also to describe who makes up the case population in terms of age and sex. These steps are important for informing the implementation of appropriate outbreak control measures in future early childhood, vaccine-preventable disease outbreaks in complex emergency and refugee settings.

## Methods

### Study Description and Population

Clinical line-list data from MdM clinics operating in Elliniko, Malakasa, and Raidestos camps in mainland Greece were used to characterize chickenpox outbreaks in this retrospective study. Data were obtained from MdM and the Centre for Research on the Epidemiology of Disasters - Association Pour l’Etude Epidemiologique des Désastres (CRED-ASED; Brussels, Belgium). This study was reviewed and determined to be exempt by the University of Delaware (Newark, Delaware USA) Institutional Review Board (2002123).

### Measures

Data were coded two distinct times by one researcher, and a single discrepancy between the codes was reconciled by a second researcher. Individuals were considered to be cases if they had a diagnosis or clinical notes indicating either confirmed or suspected chickenpox or varicella. Lab diagnostics and results were not available. Without additional information about prior vaccination status or serology for camp residents, all individuals were considered to be susceptible to chickenpox until infected. If there were multiple chickenpox diagnoses for an individual, only the first diagnosis was included in the case list. One case was excluded due to prior diagnosis within the study period. All diagnoses were cross-checked to ensure only new cases – not follow-ups – were included in the case list.

Sex was treated as a binary variable, as described by the United Nations Office for Disaster Risk Reduction’s (Geneva, Switzerland) Sex, Age, and Disability Disaggregated Data definitions.^39^ Outbreaks were considered over when there were no new cases for twice the average plausible incubation period (ie, one month).^18^ There were four distinct outbreaks - Elliniko, two distinct outbreaks in Malakasa, and Raidestos. Age was treated as a continuous variable for logistic regressions and as a four-level ordinal variable (eg, zero-to-five years; six-to-12 years; 13-19 years; and 20+ years) for age-specific incidences.

### Analysis

Median age and proportions for sex and country of origin are presented for each camp. A Kruskal-Wallis test was used to test the difference in camp and case median age. Fisher’s Exact test was used to assess the distribution of countries of origin to accommodate for small cell counts. Chi-square tests were used to compare the sex distribution of camp and case populations.

Logistic regression was used to compare the odds of chickenpox by sex, camp, and yearly increase in age. Camp-specific odds ratios and 95% confidence intervals with a priori alpha of 0.05 for age and sex are reported. Incidence rates were calculated, and epidemic curves were plotted using Microsoft Excel (Microsoft Corp.; Redmond, Washington USA). The timeline for epidemic curves starts at one-week prior to first case and ends one-month after the last recorded case.

Incidence rates were calculated using PROC GENMOD (SAS Institute; Cary, North Carolina USA) with a Poisson distribution, log link, and offset (ie, natural logarithm of person-days). Least-means squared estimation was used to generate incidence rates and 95% confidence limits.^40^ Incidences were calculated for stratified age categories and sex for each camp outbreak. All analyses were completed in SAS Studio.

## Results

Afghanistan was the largest sending country, accounting for over 72% of the population across the three camps. Syrians made up roughly 23% of the population. The remaining five percent of the population came from nine additional countries in the Middle East, South Asia, and North Africa. Males were 55.94% of the total population across the three camps. The median age of the camp populations was 19 years; ages ranged from six days to 83 years.

There was a total of 203 chickenpox cases across the camps. The largest chickenpox outbreak was in Elliniko (July 2016 - June 2017), where 6.95% of the camp population (n = 128) had chickenpox. Chickenpox outbreaks in Malakasa affected 4.07% of the population (n = 46); the first outbreak (July 2016 - November 2016) affected less than one percent of the population while the second outbreak affected 3.47% of the remaining susceptible population (November 2016 - May 2017). Nearly three percent of the population in Raidestos (n = 29; prev = 2.87%) had chickenpox from September - December 2016. Age- and sex-specific prevalences are shown in Supplemental Tables (Supplementary Material available online only).

Chickenpox cases were exclusively among Afghans in Elliniko and Malakasa and Syrians in Raidestos. Proportionally more females had chickenpox than males, although the difference in case distribution was not statistically significant (OR = 0.82; 95% CI, 0.62 - 1.09). The median age of cases across camps was five years, with the youngest case at two months and the oldest case at 41 years old.

### Elliniko

**Figure 1.**
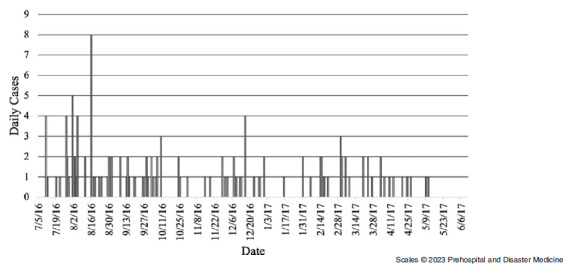
Epidemic Curve Showing Daily Chickenpox Cases in Elliniko Camp, July 2016 – June 2017.

The outbreak period for Elliniko Camp was from July 2016 - June 2017, with the last recorded case on May 11, 2017 (Figure 1). The first cases were recorded on July 11, 2016, and a total of 128 cases were recorded throughout the outbreak period. The sex distribution of cases was significantly different than the sex distribution of the entire camp population in Elliniko, with a higher sex-specific proportion of cases among females despite males making up a majority of the camp’s population (Table 1). All cases in Elliniko were among Afghans, who accounted for nearly 97% of the camp’s total population through the study period. The median age of cases in Elliniko was five years, appreciably younger than the median of 19 years of age for the camp’s population at large. The youngest case was two months old, and the oldest case was 41 years old.

**Table 1.** Descriptive Statistics for Total and Case Population of Elliniko Camp

		Elliniko Camp		Elliniko Cases		P Value
		Median	IQR	Median	IQR	
Age (median)		19	7, 30	5	3, 8.5	< .0001^a^
		**n**	**%**	**n**	**%**	
Sex	Female	777	42.21%	66	48.44%	.03^b^
	Male	1064	57.59%	62	51.56%	
Country of Origin	Afghanistan	1785	96.96%	128	100.00%	.59^c^
	Iran	10	0.54%	.	.	
	Morocco	4	0.22%	.	.	
	Pakistan	35	1.90%	.	.	
	Somalia	2	0.11%	.	.	
	Syria	5	0.27%	.	.	

a Kruskal Wallis χ^2^.

b χ^2^.

c Fisher’s Exact Test.

The overall incidence of chickenpox in Elliniko was 0.21 cases per 1,000-person-days (Table 2). The age-specific incidence of chickenpox was highest for children under 12 and for females. For children five years and younger in Elliniko, there were 0.59 cases per 1,000-person-days (IR = 0.58; 95% CI, 0.44 - 0.79), and there were 0.26 cases per 1,000-person-days for females in the camp (IR = 0.21; 95% CI, 0.18 - 0.25).

**Table 2.** Age and Sex-Specific Incidence Rates (IR) per 1,000-Person-Days for Elliniko, July 2016 – June 2017

		IR	95% CI	
Age	0-5 Years	0.58	0.46	0.74
	6-12 Years	0.59	0.44	0.79
	13-19 Years	0.08	0.04	0.16
	20+ Years	0.03	0.02	0.06
Sex	Female	0.26	0.21	0.34
	Male	0.18	0.14	0.23
Overall		0.21	0.18	0.25

### Malakasa

There were two distinct chickenpox outbreaks in Malakasa. The first outbreak of seven cases occurred from July - November 2016, with the first case recorded July 29, 2016 and the last case recorded October 11, 2016. The second outbreak occurred from November 2016 - May 2017; the first cases were recorded on November 28, 2016 and the last of 39 cases were recorded on April 18, 2017.

Among all cases across both outbreaks in Malakasa, cases were evenly distributed among men and women, with no significant difference in the distribution of cases compared to the general camp population (Table 3). The median age of cases was four years, with the youngest case was two months old and the oldest case 26 years old. All cases in Malakasa were from Afghanistan, with no cases among individuals from the five other countries of origin in the camp.

**Table 3.** Descriptive Statistics for Total and Total Case Population of Malakasa Camp

		Malakasa Camp		Malakasa Cases		P Value
		Median	IQR	Median	IQR	
Age (median)		19	8, 31	4	2, 9	< .0001^a^
		**n**	**%**	**n**	**%**	
Sex	Female	502	44.42%	23	50.00%	.44^b^
	Male	628	55.58%	23	50.00%	
Country of Origin	Afghanistan	1087	96.19%	46	100.00%	.55^c^
	Egypt	3	0.27%	.	.	
	Iran	36	3.19%	.	.	
	Pakistan	2	0.18%	.	.	
	Somalia	2	0.18%	.	.	

Note: Cases include those in both outbreaks.

a Kruskal Wallis χ^2^.

b χ^2^.

c Fisher’s Exact Test.

**Figure 2.**
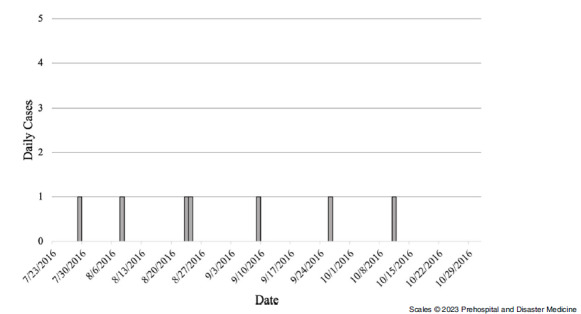
Epidemic Curve Showing Daily Chickenpox Cases in First Malakasa Camp Outbreak, July 2016 – November 2016.

**Figure 3.**
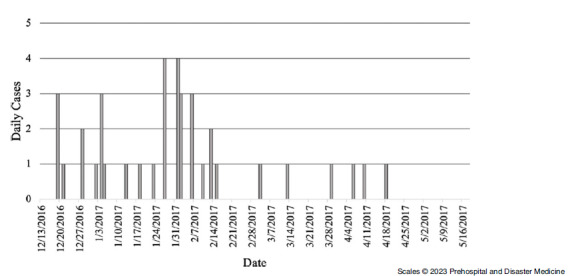
Epidemic Curve Showing Daily Chickenpox Cases in Second Malakasa Camp Outbreak, November 2016 – May 2017.

Single cases were recorded throughout the first outbreak period in Malakasa (Figure 2). The median age of cases was five years with cases ranging from one to 26 years of age. In the second outbreak, the highest number of daily cases recorded was four cases (Figure 3). The median age of cases was four years. The youngest case was two months old and the oldest was 16 years old. Just over one-half of the 39 total cases were male (n = 20; 51.28%).

The overall incidence of chickenpox in the first and smaller outbreak in Malakasa was 0.37 cases per 1,000-person-days (IR = 0.37; 95% CI, 0.28 - 0.49); Table 4. In the first outbreak, children five and under had the highest incidence of chickenpox, with 1.15 cases per 1,000-person-days (IR = 1.15; 95% CI, 0.80 - 1.66). The incidence was higher among females than males, with 0.41 cases per 1,000-person-days (IR = 0.41; 95% CI, 0.28 - 0.62). For the second outbreak (November 2016 - May 2017), the incidence was again highest among children five years and younger (IR = 0.67; 95% CI, 0.46 - 1.00). The incidence of chickenpox for females was 0.22 cases per 1,000-person-days (IR = 0.22; 95% CI, 0.14 - 0.35).

**Table 4.** Age and Sex-Specific Incidence Rates (IR) per 1,000-Person-Days for Malakasa Camp Outbreaks, July 2016 – November 2016 and November 2016 – May 2017

		Malakasa, Outbreak 1			Malakasa, Outbreak 2		
		IR	95% CI		IR	95% CI	
Age	0-5 Years	1.15	0.80	1.66	0.67	0.46	1.00
	6-12 Years	0.70	0.40	1.20	0.42	0.24	0.75
	13-19 Years	0.16	0.05	0.48	0.07	0.02	0.26
	20+ Years	0.02	0.00	0.11	0.00	0.00	0.00
Sex	Female	0.41	0.28	0.62	0.22	0.14	0.35
	Male	0.33	0.22	0.50	0.18	0.12	0.29
Overall		0.37	0.28	0.49	0.20	0.15	0.27

### Raidestos

**Figure 4.**
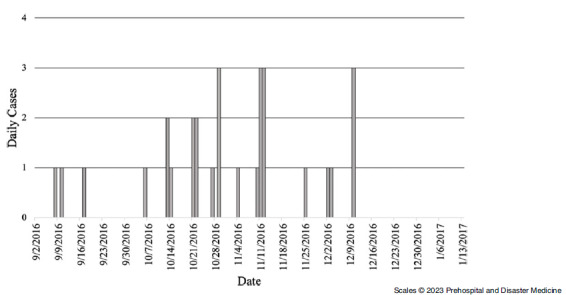
Epidemic Curve Showing Daily Chickenpox Cases in Raidestos Camp, September 2016 – December 2016.

The first chickenpox case in Raidestos Camp was recorded September 8, 2016, and the last cases were recorded December 10, 2016 (Figure 4). Throughout the outbreak period, there were 29 cases in Raidestos Camp. The median age of cases in Raidestos camp was five years compared to the camp’s median age of 19 years (Table 5). The youngest case was six months, and the oldest case was 24 years old. The majority of cases were among females. The distribution of sex within the camp and among cases were not appreciably different. All cases were from Syria. The highest number of daily cases was three, with a total of 29 cases throughout the study period.

**Table 5.** Descriptive Statistics for Total and Case Population of Raidestos Camp

		Raidestos Camp		Raidestos Cases		P Value
		Median	IQR	Median	IQR	
Age (median)		19	7, 31	5	2, 7	< .0001^a^
		**n**	**%**	**n**	**%**	
Sex	Female	475	47.03%	19	65.52%	.17^b^
	Male	535	40.59%	10	34.48%	
Country of Origin	Afghanistan	2	0.20%	.	.	.67^c^
	Algeria	4	0.40%	.	.	
	Egypt	.	.	.	.	
	Iran	4	0.40%	.	.	
	Iraq and Kurdistan	74	7.33%	.	.	
	Libya	3	0.30%	.	.	
	Morocco	2	0.20%	.	.	
	Pakistan	11	1.09%	.	.	
	Occ. Palestinian Ter.	5	0.50%	.	.	
	Somalia	1	0.10%	.	.	
	Syria	904	89.50%	29	100.00%	

a Kruskal Wallis χ^2^.

b χ^2^.

c Fisher’s Exact Test.

There were 0.22 cases per 1,000-person-days among refugees in Raidestos camp (Table 6). The age-specific incidence was highest among children five and under (IR = 0.65; 95% CI, 0.40 - 1.04), followed by children six to twelve years (IR = 0.40; 95%, CI 0.21 - 0.77). The sex-specific incidence of chickenpox was higher among males, with 0.27 cases per 1,000-person-days (IR = 0.27; 95% CI, 0.17 - 0.43).

**Table 6.** Age and Sex-Specific Incidence Rates (IR) per 1,000-Person-Days for Raidestos Camp, September 2016 – December 2016

		IR	95% CI	
Age	0-5 Years	0.65	0.40	1.04
	6-12 Years	0.40	0.21	0.77
	13-19 Years	0.12	0.03	0.46
	20+ Years	0.02	0.00	0.11
Sex	Female	0.16	0.09	0.30
	Male	0.27	0.17	0.43
Overall		0.22	0.15	0.32

### Comparing Camps

**Figure 5.**
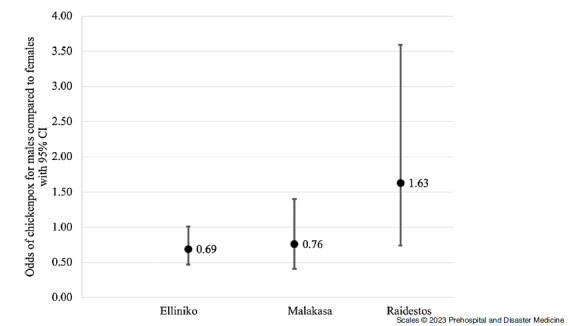
Sex-Specific Odds of Chickenpox with 95% Confidence Intervals, Comparing Males and Females.

**Figure 6.**
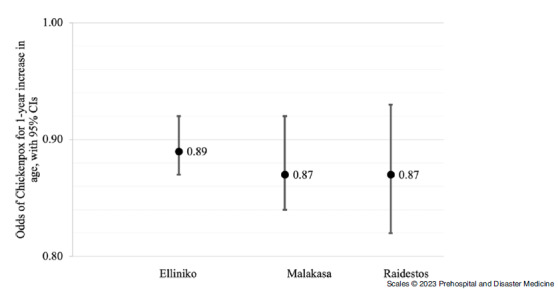
Odds of Chickenpox for One Year Increase in Age with 95% Confidence Intervals.

Sex- and age-specific odds of chickenpox are summarized in Figure 5 and Figure 6. When controlling for sex, the odds of chickenpox decreased in all three camps. In Elliniko, it decreased by 11% with each additional year of age (OR = 0.89; 95% CI, 0.87 - 0.92) and by 13% in both Malakasa (OR = 0.87; 95% CI, 0.84 - 0.92) and Raidestos (OR = 0.87; 95% CI, 0.82 - 0.93) with each additional year of age. In Raidestos, the odds of chickenpox were 63% higher for males than females when controlling for age (OR = 1.63; 95% CI, 0.74 - 3.59). However, chickenpox was more common among females than males in Elliniko (OR = 0.69; 95% CI, 0.48 - 1.01) and Malakasa camps (OR = 0.76; 95% CI, 0.84 - 0.92), with the odds of chickenpox 31% and 24% lower, respectively, among males when controlling for age.

When controlling for sex and age, the odds of chickenpox were 2.74-times higher among individuals at Elliniko than Raidestos (OR = 2.74; 95% CI, 1.80 - 4.17). The odds of chickenpox at Malakasa and Raidestos were not appreciably different when controlling for age and sex, with those at Malakasa having 50% higher odds of chickenpox than at Raidestos (OR = 1.49; 95% CI, 0.92 - 2.42). Individuals at Malakasa were 46% less likely to have chickenpox than those at Elliniko (OR = 0.46; 95% CI, 0.38 - 0.78).

## Discussion

The median age of cases was five years for Elliniko and Raidestos and four years for Malakasa. The age-specific incidence of chickenpox across camps was highest among primary school aged children (eg, 12 years and under). While the median ages were four to five years, nearly 45% of cases across the camps were over the age of five, and roughly 12% of cases were 13 years or older. These findings reflect expected age-specific characteristics of chickenpox outbreaks and support the inclusion of older children, adolescents, and young adults in immunization activities among displaced populations.

The relationship between immunity with country of origin and age was explored in a study among asylum seekers in Lower Saxony, Germany in 2015.^41^ The current findings reflect the lower levels of immunity in Afghans and higher immunity with increasing age presented by Toikkanen, et al. In their study, only Syrians had population-level seroprevalence of antibodies for chickenpox that indicated they had reached the herd immunity of 91%.^41^ As a whole, Afghans fell short of herd immunity levels, with those aged 12-29 having seroprevalences of varicella-specific immunoglobulins lower than 91%. However, the odds of immunity as determined by serology increased four percent for every additional year of age. This finding is reflected in the current study, although with a different measure of presumed immunity, where the odds of being a chickenpox case decreased by 11% (Elliniko) and 13% (Malakasa and Raidestos) for every additional year in age, indicating higher odds of immunity as age increases.

Despite comprising a comparatively lower proportion of the total camp population, females were more likely to have chickenpox than males in both Elliniko and Malakasa camps when controlling for age; incidence followed the same patterns. There were seven cases of chickenpox among women of child-bearing age (eg, 15-45 years). Although a proportionally small share of overall cases, the health risks to both mother and fetus due to chickenpox infection warrant particular attention for this demographic.^42^ While males and females are at equal risk for contracting chickenpox following exposure, sociocultural differences in exposure rates and timely access to treatment for cases between males and females in camp settings should be further explored to better understand both risk and incidence of chickenpox and other respiratory pathogens in refugee camps.

### Seasonality

In Elliniko, cases were consistent throughout the year, with the outbreak period extending from July 2016 - June 2017. While cases tend to be inversely related with temperature, even in temperate climate zones, consistent caseloads throughout the year are more commonly experience in temperate climate zones relative to tropical zones.^29–31^ In contrast to the Elliniko outbreak, the larger of two outbreaks in Malakasa camp and the outbreak in Raidestos reflect expected seasonality of chickenpox, with higher incidence of cases in cooler seasons. Specifically, the larger of two outbreaks in Malakasa camp occurred from December 2016 - May 2017, with case counts staying consistently lower after February 2017. The outbreak in Raidestos started in September 2016, with a peak in cases in November.

Demographic flows can contribute to deviations from expected seasonal patterns of disease. Flows account for both in-flows of new refugees and out-flows due to resettlement, repatriation, naturalization, and death, both of which can contribute to both social and demographic changes in camps.^43^ The consistent flows in refugee camps in Greece could contribute to the examples in the Elliniko and Malakasa outbreaks, which were persistent over time rather than showing distinct cooler weather seasonality.^44^ This work shows that deviances from expected seasonal patterns are plausible in complex emergencies, and the influence of instability, population mobility, and other environmental drivers on climatic drivers of seasonality should be further investigated in camp settings and considered as part of vaccine prioritization. However, outbreaks may align with expected seasonality, providing useful information for ensuring timely and equitable outbreak preparedness and response activities.

### Camp Circumstances and Chickenpox Outbreaks

Toikkanen, et al set a herd immunity threshold of 91% for refugees and asylum seekers 12 years and older that were screened for antibodies against varicella upon arrival in Lower Saxony, Germany. This threshold was likely conservative, especially given crowding, ventilation, and other environmental factors in refugee camps and in-take facilities.^41^ The authors suggested that, despite reasonably high levels of immunity at the population-level among refugees and asylum seekers in Lower Saxony, adolescents and adults needed to be included in immunization activities to maximize both individual- and population-level protection.

The risk factors noted by Toikkanen, et al - crowding, poor ventilation, and insufficient facilities - were also present in all three camps. Camp settings, despite the best efforts of governmental, non-governmental, and humanitarian actors, are often dire, creating environments that are primed for communicable disease outbreaks. For the current study, there was no information available regarding steps taken to isolate cases and implement infection control measures. The odds of chickenpox were highest in Elliniko, the largest of the three camps. The odds of chickenpox were comparable between Malakasa and Raidestos. For Elliniko specifically, limits on hygiene facilities, as well as inadequate shelter, could have contributed to persistent cases throughout the study period. Efforts to ensure adequate accommodations as outbreak control measures would be further supported by presumptive immunization for new arrivals against varicella.

## Limitations

Data used in this study only include individuals receiving primary health care consultations at MdM clinics. Cases of chickenpox were clinician diagnosed, and there was no lab confirmation information available. Therefore, presumptive cases were included in the analysis, although there could have been differential diagnoses. Information on individual immunization status and time in camp were also not available. Missing data in the form of missed or undiagnosed cases could bias the estimates presented in this study, likely resulting in under-estimates of true chickenpox incidence. Because this study uses clinical line-list data, it is highly likely that there were unidentified cases for individuals who were not seen MdM clinics for various reasons, including receiving consultations via other providers or having sub-clinical cases.

All individuals were considered to be susceptible to chickenpox until infected. While less than five percent of adults are estimated to be susceptible to chickenpox in temperate climates,^45^ all individuals in camps were considered to be susceptible. This inflated the total number of susceptible individuals in camps; therefore, it is highly probable that incidence was under-estimated. The decision to consider all individuals to be susceptible reflects that camp populations are disproportionately younger than the general population. Additionally, susceptibility among refugees and displaced persons can be exacerbated due to impaired immune function resulting from persistent stressors;^46–48^ differences in demographics and natural history of disease;^49^ crowded and unhygienic living conditions;^50^ and limited access to health care among refugees in transit countries like Greece.^51–54^ Further work should investigate the role that these risk factors play in outbreaks among displaced camp populations. Additionally, future studies should explore long-term disease sequelae to better understand morbidity and mortality among high-risk camp populations.

## Conclusions

In this study, the incidence of chickenpox was highest among primary school aged children (eg, 12 and under), as expected. There were no differences in incidence by sex. Identifying the age- and sex-dynamics of outbreaks – and potential deviations from expected patterns – is important to ensure the implementation of adequate, appropriate, and targeted control measures. The importance of this is even more pronounced among populations who possess different risks for infection, including refugees with impaired immune function from persistent stressors related to travel, accommodations, and health care access.

## Supplementary material

For supplementary material accompanying this paper visit https://doi.org/10.1017/S1049023X23006702. click here to view supplementary material
