# Supplementary material for: Chickenpox Outbreaks in Three Refugee Camps on Mainland Greece, 2016-2017: A Retrospective Study
Source: Prehosp Disaster Med. 2023 Dec 18;39(1):3–12. doi: 10.1017/S1049023X23006702 (PMC10882556; doi:10.1017/S1049023X23006702)
Supplement: Supplementary file 1 [file S1049023X23006702sup001.docx]

**Supplemental Table 1.** Age stratified prevalences of chickenpox for Elliniko.

| AGE | Cases | Total | Prev |
| --- | --- | --- | --- |
| 0 - 5 y | 66 | 375 | 17.60% |
| 6 - 12 y | 44 | 248 | 17.74% |
| 13 - 19 y | 8 | 303 | 2.64% |
| 20 + y | 10 | 915 | 1.09% |
| Total | 128 | 1841 | 6.95% |

**Supplemental Table 2.** Age stratified prevalences of chickenpox for Malakasa.

| AGE | Malakasa, Outbreak 1 | | | Malakasa, Outbreak 2 | | |
| --- | --- | --- | --- | --- | --- | --- |
|  | Cases | Total | Prev | Cases | Total | Prev |
| 0 - 5 y | 4 | 229 | 1.75% | 25 | 225 | 11.11% |
| 6 - 12 y | 1 | 169 | 0.59% | 12 | 168 | 7.14% |
| 13 - 19 y | 1 | 175 | 0.57% | 2 | 174 | 1.15% |
| 20 + y | 1 | 557 | 0.18% | 0 | 556 | 0.00% |
| Total | 7 | 1130 | 0.62% | 39 | 1123 | 3.47% |

**Supplemental Table 3.** Age stratified prevalences of chickenpox for Raidestos.

| AGE | Cases | Total | Prev |
| --- | --- | --- | --- |
| 0 - 5 y | 17 | 208 | 8.17% |
| 6 - 12 y | 9 | 174 | 5.17% |
| 13 - 19 y | 2 | 131 | 1.53% |
| 20 + y | 1 | 497 | 0.20% |
| Total | 29 | 1010 | 2.87% |

**Supplemental Table 4.** Sex stratified prevalences of chickenpox across Elliniko.

| SEX | Cases | Total | Prev |  |
| --- | --- | --- | --- | --- |
| Females | 66 | 777 | 8.49% | |
| Males | 62 | 1064 | 5.83% | |
| Total | 128 | 1841 | 6.95% | |

**Supplemental Table 5.** Sex stratified prevalences of chickenpox across Malakasa.

| SEX | Malakasa, Outbreak 1 | | | Malakasa, Outbreak 2 | | |
| --- | --- | --- | --- | --- | --- | --- |
|  | Cases | Total | Prev | Cases | Total | Prev |
| Females | 4 | 502 | 0.80% | 19 | 498 | 3.82% |
| Males | 3 | 628 | 0.48% | 20 | 625 | 3.20% |
| Total | 7 | 1130 | 0.62% | 39 | 1123 | 3.47% |

**Supplemental Table 6.** Sex stratified prevalences of chickenpox across Malakasa.

| SEX | Cases | Total | | Prev |
| --- | --- | --- | --- | --- |
| Females | 10 | | 475 | 2.11% |
| Males | 19 | | 535 | 3.55% |
| Total | 29 | | 1010 | 2.87% |

**Supplemental Table 7.** Age-sex stratified prevalences of chickenpox for females Elliniko.

| AGE FEMALES | Cases | Total | Prev |
| --- | --- | --- | --- |
| 0 - 5 y | 33 | 173 | 19.08% |
| 6 - 12 y | 25 | 105 | 23.81% |
| 13 - 19 y | 2 | 106 | 1.89% |
| 20 + y | 6 | 393 | 1.53% |
| Total | 66 | 777 | 8.49% |

**Supplemental Table 8.** Age-sex stratified prevalences of chickenpox for females Malakasa.

| AGE FEMALES | Malakasa, Outbreak 1 | | | Malakasa, Outbreak 2 | | |
| --- | --- | --- | --- | --- | --- | --- |
|  | Cases | Total | Prev | Cases | Total | Prev |
| 0 - 5 y | 1 | 103 | 0.97% | 12 | 102 | 11.76% |
| 6 - 12 y | 1 | 78 | 1.28% | 7 | 77 | 9.09% |
| 13 - 19 y | 1 | 55 | 1.82% | 0 | 54 | 0.00% |
| 20 + y | 1 | 266 | 0.38% | 0 | 265 | 0.00% |
| Total | 4 | 502 | 0.80% | 19 | 498 | 3.82% |

**Supplemental Table 5.** Age-sex stratified prevalences of chickenpox for females Raidestos.

| AGE FEMALES | Cases | Total | Prev |
| --- | --- | --- | --- |
| 0 - 5 y | 6 | 94 | 6.38% |
| 6 - 12 y | 4 | 72 | 5.56% |
| 13 - 19 y | 0 | 64 | 0.00% |
| 20 + y | 0 | 245 | 0.00% |
| Total | 10 | 475 | 2.11% |

**Supplemental Table 9.** Age-sex stratified prevalences of chickenpox for males in Elliniko.

| AGE MALES | Cases | Total | Prev |
| --- | --- | --- | --- |
| 0 - 5 y | 33 | 202 | 16.34% |
| 6 - 12 y | 19 | 143 | 13.29% |
| 13 - 19 y | 6 | 197 | 3.05% |
| 20 + y | 4 | 522 | 0.77% |
| Total | 62 | 1064 | 5.83% |

**Supplemental Table 10.** Age-sex stratified prevalences of chickenpox for males in Malakasa.

| AGE MALES | Malakasa, Outbreak 1 | | | Malakasa, Outbreak 2 | | |
| --- | --- | --- | --- | --- | --- | --- |
|  | Cases | Total | Prev | Cases | Total | Prev |
| 0 - 5 y | 3 | 126 | 2.38% | 13 | 123 | 10.57% |
| 6 - 12 y | 0 | 91 | 0.00% | 5 | 91 | 5.49% |
| 13 - 19 y | 0 | 120 | 0.00% | 2 | 120 | 1.67% |
| 20 + y | 0 | 291 | 0.00% | 0 | 291 | 0.00% |
| Total | 3 | 648 | 0.46% | 20 | 625 | 3.20% |

**Supplemental Table 11.** Age-sex stratified prevalences of chickenpox for males in Raidestos.

| AGE MALES | Cases | Total | Prev |
| --- | --- | --- | --- |
| 0 - 5 y | 11 | 114 | 9.65% |
| 6 - 12 y | 5 | 102 | 4.90% |
| 13 - 19 y | 2 | 67 | 2.99% |
| 20 + y | 1 | 252 | 0.40% |
| Total | 19 | 535 | 3.55% |
